# Draft Genome Sequence of Xanthomonas arboricola Strain 3004, a Causal Agent of Bacterial Disease on Barley

**DOI:** 10.1128/genomeA.01572-14

**Published:** 2015-02-19

**Authors:** Alexander N. Ignatov, Elena I. Kyrova, Svetlana V. Vinogradova, Anastasia M. Kamionskaya, Norman W. Schaad, Douglas G. Luster

**Affiliations:** a Center Bioengineering, Russian Academy of Sciences, Moscow, Russian Federation; b Russian Research Institute of Phytopathology, Bolshie Vyazemy, Russian Federation; c Peoples’ Friendship University of Russia, Moscow, Russia Federation; d U.S. Department of Agriculture, Agricultural Research Service, Foreign Disease–Weed Science Research Unit, Ft. Detrick, Maryland, USA

## Abstract

We report here the annotated genome sequence of Xanthomonas arboricola strain 3004, isolated from barley leaves with symptoms of streak and capable of infecting other plant species. We sequenced the genome of X. arboricola strain 3004 to improve the understanding of molecular mechanisms of the pathogenesis and evolution of the genus Xanthomonas.

## GENOME ANNOUNCEMENT

Xanthomonas arboricola is a species of phytopathogenic bacteria characterized by virulence specificity to plural host plant species (1). For example, strains of X. arboricola have been isolated from diseased plants of the families *Rosaceae*, *Juglandaceae*, *Betulaceae*, *Salicaceae*, *Musáceae*, *Euphorbiaceae*, *Brassicaceae*, *Poaceae*, and *Solanaceae* (1–6). A collection of 32 strains identified as X. arboricola by virulence and specific biochemical and molecular tests, and obtained from diseased plants of 4 different families originating from the Russian Federation, was studied by multilocus sequence type (MLST) analysis as described previously (2, 3). It has been shown that the strains had alleles of at least one of several genes more similar to strains of other xanthomonads (7). To investigate the hypothesis of putative horizontal gene transfer between the species of Xanthomonas, we determined the draft genome sequence of Xanthomonas arboricola strain 3004, isolated from barley plants, with virulence to plants of barley, brassicas, and chestnut. The genome of strain 3004 was sequenced using the Roche GS FLX pyrosequencing platform. Sequencing was performed using a whole-genome strategy employing shotgun and paired-end genome libraries (178.5 Mb). Shotgun and paired-end reads were assembled into 132 contigs from 528 to 98,173 bp by GS *de novo* assembler version 2.3 (454 Life Sciences, Branford, CT, USA). The *N*_50_ is 61,665 bp. The total size of the assembled genome is 4,765,897 bp (GC content, 65.3%), which is smaller than that of other sequenced X. arboricola strains (8–10).

The annotation for the strain 3004 genome sequence was conducted using the RAST server (11) and detected 4,113 coding sequences, 2 rRNAs, and 55 RNAs, representing 450 subsystems, which is similar to the 3,912–4,500 coding sequences in 436 to 449 subsystems for other X. arboricola strains (8–10). 16S rRNA comparison analysis performed with BLASTn (12) revealed 100% identity to other X. arboricola strains. Furthermore, in order to check the robustness of the genome sequence of strain 3004, a MLST analysis using genes *gyrB*, *dnaK*, *rpoD*, *nrdB*, *prpC*, *fabB*, and *purA* clustered strain 3004 into the X. arboricola phylogroup. Whole-genome nucleotide comparison against the draft genome sequence of strain 3004 indicated >99% identity to the draft genome sequences of X. arboricola pv. *selebensis* strains NCPPB 1832 and NCPPB 1630. We have not found sequences of type 3 secretion system (T3SS) genes nor the 53 effector protein genes (T3E) found in other xanthomonads (13). PCR with T3E-specific primers (13, 14) using genomic DNA did not result in amplification of strain 3004, as well as nearly all of the tested 32 strains of X. arboricola from the Russian Federation, whereas expected bands were detected in the positive controls selected from different Xanthomonas species. The genome information presented here will allow comparative studies with other X. arboricola pathovars to help in understanding the molecular mechanisms behind plant-pathogen interactions.

### Nucleotide sequence accession numbers.

The whole-genome shotgun project of X. arboricola 3004 has been deposited in GenBank under the accession number AZQY00000000. The version described in this paper is the first version, AZQY00000000.1.
